# Neural pathway activation in the subthalamic region depends on stimulation polarity

**DOI:** 10.1093/braincomms/fcaf006

**Published:** 2025-01-21

**Authors:** Seyyed Bahram Borgheai, Enrico Opri, Faical Isbaine, Eric R Cole, Roohollah Jafari Deligani, Nealen G Laxpati, Benjamin B Risk, Jon T Willie, Robert E Gross, Nicholas AuYong, Cameron C McIntyre, Svjetlana Miocinovic

**Affiliations:** Department of Neurology, Emory University, Atlanta, GA 30322, USA; Department of Biomedical Engineering, University of Michigan, Ann Arbor, MI 48109, USA; Department of Neurosurgery, Emory University, Atlanta, GA 30322, USA; Department of Biomedical Engineering, Emory University and Georgia Institute of Technology, Atlanta, GA 30332, USA; Department of Neurology, Emory University, Atlanta, GA 30322, USA; Department of Neurosurgery, Emory University, Atlanta, GA 30322, USA; Department of Biostatistics and Bioinformatics, Emory University, Atlanta, GA 30322, USA; Department of Neurological Surgery, Washington University School of Medicine, St Louis, MO 63110USA; Department of Neurosurgery, Emory University, Atlanta, GA 30322, USA; Department of Neurosurgery, Rutgers Robert Wood Johnson Medical School, New Brunswick, NJ 08901, USA; Department of Neurosurgery, Emory University, Atlanta, GA 30322, USA; Department of Biomedical Engineering, Emory University and Georgia Institute of Technology, Atlanta, GA 30332, USA; Department of Cell Biology, Emory University, Atlanta, GA 30322, USA; Department of Biomedical Engineering, Duke University, Durham, NC 27708, USA; Department of Neurology, Emory University, Atlanta, GA 30322, USA; Department of Biomedical Engineering, Emory University and Georgia Institute of Technology, Atlanta, GA 30332, USA

**Keywords:** hyperdirect pathway, electrocorticography, chronaxie, anodic, evoked resonant neural activity

## Abstract

Deep brain stimulation (DBS) is an effective treatment for Parkinson’s disease; however, there is limited understanding of which subthalamic pathways are recruited in response to stimulation. Here, by focusing on the polarity of the stimulus waveform (cathodic versus anodic), our goal was to elucidate biophysical mechanisms that underlie electrical stimulation in the human brain. In clinical studies, cathodic stimulation more easily triggers behavioural responses, but anodic DBS broadens the therapeutic window. This suggests that neural pathways involved respond preferentially depending on stimulus polarity. To experimentally compare the activation of therapeutically relevant pathways during cathodic and anodic subthalamic nucleus (STN) DBS, pathway activation was quantified by measuring evoked potentials resulting from antidromic or orthodromic activation in 15 Parkinson’s disease patients undergoing DBS implantation. Cortical evoked potentials (cEPs) were recorded using subdural electrocorticography, DBS local evoked potentials (DLEPs) were recorded from non-stimulating contacts, and electromyography activity was recorded from arm and face muscles. We measured (i) the amplitude of short-latency cEP, previously demonstrated to reflect activation of the cortico-STN hyperdirect pathway, (ii) DLEP amplitude thought to reflect activation of STN-globus pallidus (GP) pathway and (iii) amplitudes of very short-latency cEPs and motor evoked potentials for activation of corticospinal/bulbar tract (CSBT). We constructed recruitment and strength–duration curves for each EP/pathway to compare the excitability for different stimulation polarities. We compared experimental data with the most advanced DBS computational models. Our results provide experimental evidence that subcortical cathodic and anodic stimulation activate the same pathways in the STN region and that cathodic stimulation is in general more efficient. However, relative efficiency varies for different pathways so that anodic stimulation is the least efficient in activating CSBT, more efficient in activating the hyperdirect pathway and as efficient as cathodic in activating STN-GP pathway. Our experiments confirm biophysical model predictions regarding neural activations in the central nervous system and provide evidence that stimulus polarity has differential effects on passing axons, terminal synapses, and local neurons. Comparison of experimental results with clinical DBS studies provides further evidence that the hyperdirect pathway may be involved in the therapeutic mechanisms of DBS.

## Teaser

Borgheai *et al*. have quantified activation of neural pathways in subthalamic region by recording potentials evoked by cathodic or anodic deep brain stimulation in patients with Parkinson’s disease. They conclude that relative activation efficiency varies between pathways. They relate these findings to fundamental biophysical principles, computational models and clinical studies.

## Graphical Abstract

**Graphical Abstract:**
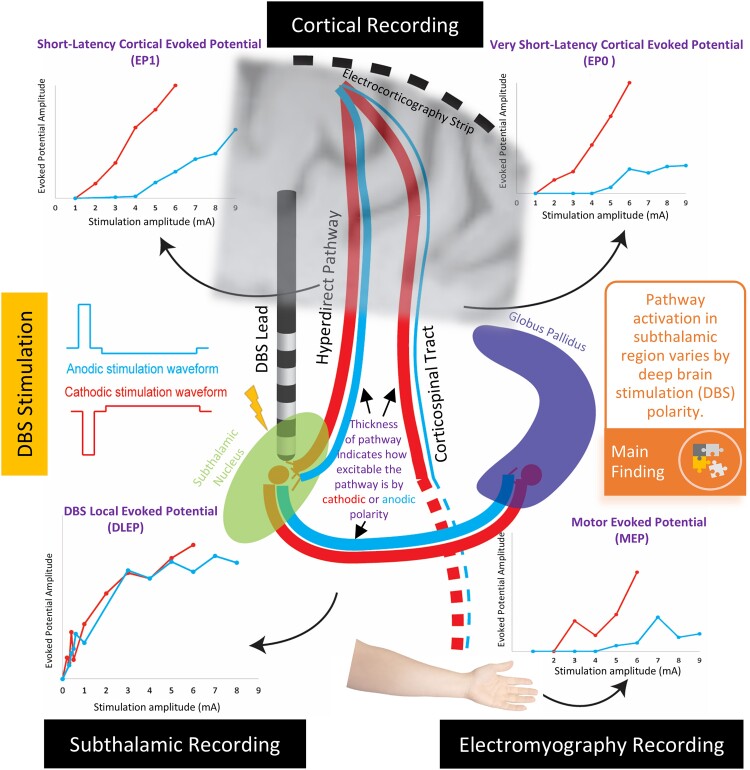

See Asadi, Koirala, and Muthuraman (https://doi.org/10.1093/braincomms/fcaf061) for a scientific commentary on this article.

## Introduction

Selective modulation of neurons with electrical stimulation is desirable in clinical neuromodulation applications where the aim is to maximize target engagement while limiting off-target effects, but this is difficult to achieve. Despite the effectiveness and relative safety of deep brain stimulation (DBS) therapy for medication-refractory Parkinson's disease being established in multiple clinical trials,^1-3^ how stimulation parameters impact recruitment of clinically relevant pathways in the subthalamic region is still not well understood.

In particular, little is known about the effect of stimulus waveform polarity, i.e. cathodic versus anodic, or their underlying neurophysiological actions. First-generation DBS devices were restricted to cathodic pulses during monopolar stimulation by always utilizing the implanted pulse generator case as the anode. This configuration was supported by clinical and pre-clinical data demonstrating that monopolar cathodic stimulation elicits behavioural responses more readily than anodic stimulation.^4,5^ However, recent clinical studies have demonstrated that anodic DBS can widen the therapeutic window, a difference in a stimulation parameter value that results in therapeutic benefit versus unwanted side effect.^6,7^ Putative pathways responsible for DBS therapeutic benefit include the subthalamopallidal [subthalamic nucleus-globus pallidus (STN-GP)] projections^8^ and cortico-subthalamic hyperdirect pathway (HDP),^9,10^ while motor side effects (unwanted muscle contractions) are due to activation of the corticospinal and corticobulbar tracts (CSBT).^11^ Computational studies have suggested that anodic DBS may be more efficient (lower activation threshold) than cathodic when activating terminating axons of the HDP compared with passing axons of the CSBT^12-14^; however, relative activation thresholds also depend on neuronal morphology.^15^ When coupled with fundamental biophysical knowledge, differential responses to cathodic and anodic stimulation can provide novel insights into which neuronal elements are activated by DBS.

**Figure 1:**
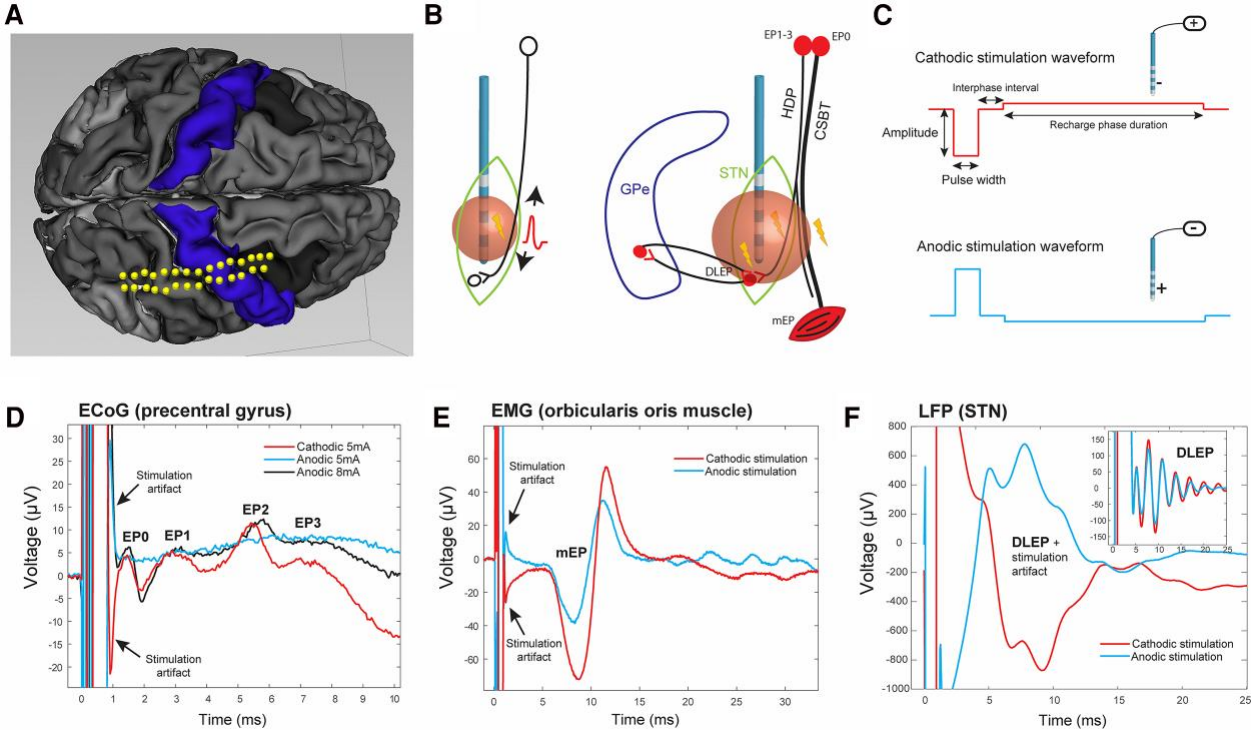
**Experimental setup. (A)** ECoG strip, co-registered with surface-extracted preoperative MRI, was placed over the arm area of the primary motor cortex. **(B)** Left: activation of an axon during extracellular stimulation results in antidromic and orthodromic action potential propagation. Right: a schematic of clinically relevant pathways in the STN region and evoked potentials corresponding to their activation. **(C)** Asymmetric biphasic waveform for monopolar cathodic and anodic stimulation (see text for details). **(D–F)** Examples of recorded evoked potentials during cathodic and paired anodic stimulation for cEPs, including very short-latency peak (EP0; <2 ms) and short-latency peaks (EP1–3; 2–10 ms) **(D)**, mEP **(E)** and raw and filtered (inset box) DLEP **(F)**. Each trace was obtained by averaging signal in response to ∼120 DBS pulses. Stimulation artefact reverses direction while neural or muscle response is consistent for cathodic and anodic stimulation. cEP, cortical evoked potential; CSBT, corticospinal/bulbar tract; DLEP, DBS local evoked potential; ECoG, electrocorticography; EMG, electromyography; GPe, globus pallidus externa; HDP, hyperdirect pathway; LFP, local field potential; mEP, motor evoked potential; STN, subthalamic nucleus.

Experimental investigations have long sought to dissect the activation of DBS-relevant pathways, albeit almost exclusively with traditional cathodic stimulation. The short-latency (2–10 ms) cortical evoked potentials (cEPs) recorded over the sensorimotor and prefrontal regions in response to single STN DBS pulses have been characterized as markers of antidromic activation of the HDP.^16-23^ A previous intraoperative study identified three short-latency evoked potentials (EP1–3) over the sensorimotor cortex as related to antidromic HDP activation, with EP1 being related to therapeutic stimulation (Fig. 1D).^17^ The very short-latency cortical potentials (<2 ms; EP0) have been associated with CSBT activation (Fig. 1D) and accompanied by motor evoked potentials (mEPs) recorded peripherally in muscles (Fig. 1E).^17^ The DBS-related mEPs have been reported by several other investigators to be associated with DBS-induced motor side effects.^24,25^ Activation of the STN-GP pathway has recently been proposed to be reflected in the DBS local evoked potentials (DLEPs) (Fig. 1F).^26^ The DLEP signal, also known as evoked resonant neural activity (ERNA), is recorded from inactive DBS lead contacts around the stimulating contact in the STN and is related to the therapeutic efficacy of DBS.^27-30^

The goal of this study was to experimentally quantify and compare the activation of DBS-relevant pathways (HDP, CSBT and STN-GP) during cathodic and anodic DBS in patients with Parkinson’s disease. We hypothesized that anodic stimulation is particularly inefficient (achieving less activation with the same amount of current) in activating the side effect-inducing CSBT compared with cathodic stimulation, and it is more efficient when activating the therapeutic HDP and STN-GP pathways (but still less efficient overall than cathodic stimulation). We constructed recruitment and strength–duration curves for each EP/pathway to compare excitability across different stimulation polarities. Each strength–duration curve was used to estimate the chronaxie, a metric closely related to the time constant of the activated neuronal element.^31^ We compare our experimental findings with computational modelling predictions to further elucidate biophysical mechanisms of electrical stimulation in the human brain and with prior clinical observations to shed light on which subthalamic pathways may be contributing to DBS therapeutic benefits.

## Materials and methods

### Patient selection

Patients with idiopathic Parkinson’s disease scheduled to undergo awake STN DBS surgery at a large academic centre were recruited for the study (Table 1). Informed consent was obtained before surgery under the protocol approved by the Institutional Review Board. All patients were informed that a temporary subdural electrocorticography (ECoG) recording electrode would be placed strictly for research purposes during surgery.

**Table 1:** Patient demographics and experimental setup

Patient code	Age/sex	Stimulated hemisphere	ECoG laterality at M1 (mm)	Number of M1/total ECoG channels	Number of face/limb EMG channels	Number of cathodic DBS settings	Number of anodic DBS settings	Number of paired DBS settings	DBS lead bottom contact MCP coordinates (mm)	DBS lead model
P01	53/M	R	28.0	9/26	3/3	39	5	5	10.7 −2.5 −6.6	BSC 2202
P02	60/M	L	26.5	10/26	1/3	34	6	4	−12.7 −2.3 −4.2	ABT 6172
P03	63/M	R	23.9	11/26	0/4	31	19	19	12.4 −2.6 −2.9	ABT 6172
P04	48/F	R	29.0	9/26	2/3	19	4	4	9.6 −3.3 −7.9	BSC 2202
P05	65/M	R	20.0	2/5	2/3	40	6	6	9.2 −3.4 −5.9	BSC 2202
P06	55/F	R	24.1	1/5	2/3	32	6	6	11.4 0.3 −4.4	BSC 2202
P07	63/M	R	27.6	10/26	3/2	8	7	7	8.1 −4.4 −5.7	BSC 2202
P08	42/M	R	22.7	3/5	0/0	4	4	4	12.1 −2 −6.5	BSC 2202
P09	64/M	R	20.9	2/5	0/3	4	4	4	12.5 −2.9 −4.7	MDT B33005
P10	43/M	L	32.5	6/26	1/4	24	24	24	−12.5 −2.6 −3.9	BSC 2022
P11	64/M	R	32.3	7/26	3/4	25	22	10	9.4 −3.6 −6.6	BSC 2022
P12	65/F	R	36.6	9/26	4/4	62	60	10	9.0 −2.5 −4.4	BSC 2022
P13	54/M	R	29.9	8/26	4/4	41	43	8	11.3 −2.8 −5.5	BSC 2022
P14	42/F	R	25.5	7/26	0/4	52	52	7	8.5 −4.0 −6.2	BSC 2022
P15	75/M	L	NA	NA	NA	40	40	32	−11.4 −3.4 −7.0	BSC 2022

ABT, Abbott; BSC, Boston Scientific; M1, primary motor cortex; MCP, mid-commissural point; MDT, Medtronic.

### Research-related surgical methods

Segmented DBS electrodes (Medtronic, Abbott or Boston Scientific models) were implanted using microelectrode guidance and standard clinical procedures.^32^ Our experiments followed the standard clinical protocol where patients were under propofol sedation for burr hole drilling, but fully awake for the intraoperative recordings. To record cEPs, an ECoG strip (28 or 6 contacts; Ad-Tech) was placed subdurally through the same burr hole used for DBS implantation.^17,33^ The 28-contact strip had two rows of fourteen 2 mm diameter platinum contacts separated by 4 mm. The 6-contact strip had one row of 4 mm diameter platinum contacts separated by 10 mm. The intended target location for the centre of the strip was the arm area of M1, ∼3 cm from the midline and slightly medial to the hand knob. The mEPs were recorded using surface electromyography (EMG) electrodes placed on the contralateral arm (biceps, flexor carpi radialis, extensor carpi radialis and first dorsal interosseous) and bilateral face (nasalis and orbicularis oris). The DBS electrode was used to deliver stimulation and to record DLEP activity from non-stimulating contacts. Recordings were performed at least 12 h after stopping all anti-parkinsonian medications and at least 30 min after stopping propofol sedation.

### Stimulation

Electrical stimulation was delivered through the DBS electrode contacts while patients were at rest using the NeuroOmega electrophysiology system (Alpha Omega Engineering). The number of stimulation settings tested varied between patients depending on the duration of time available intraoperatively (average 50, range 8–122). The stimulation settings varied in active contact, amplitude, pulse width and stimulus polarity and were delivered in a pseudorandomized order (*randperm* function in MATLAB). All stimulation settings were monopolar and a 2 × 3 inch surface electrode on the shoulder ipsilateral to the stimulated brain hemisphere was used as the return. Stimulation frequency was 10 Hz (except 9 Hz for P08) and was applied for 12 s (13.3 s for P08) with 3 s pause between the settings. The 10 Hz frequency was chosen to ensure sufficient post-stimulus time (100 ms) to be able to record all evoked potentials of interest similar to previous work.^17^ In three patients (P12, P13 and P15), stimulation settings below 1 mA were applied for 3 s with 1 s pause specifically to elicit DLEP (DLEP measurements required fewer pulses as the signal-to-noise ratio was typically high). The stimulation waveform was asymmetric biphasic with 70 µs inter-phase duration with the first, large-amplitude phase that defined stimulation polarity (cathodic or anodic) and similar to a waveform delivered by clinical stimulators.^34,35^ Details of stimulation waveforms are described in the Supplementary material.

### Signal acquisition

ECoG, local field potentials (LFPs) and EMG signals were recorded simultaneously with the NeuroOmega. Signals were amplified and acquired at 22 kHz sampling rate with a built-in hardware band-pass filtering between 0.075 and 3500 Hz. The contra- and ipsilateral ear lobes were used as the ECoG reference and ground, respectively. The EMG signals were recorded from pair-referenced electrodes and a common ground was on the contralateral knee. The LFP was recorded from all non-stimulating DBS contacts. An ipsilateral scalp needle was used as a recording reference while corresponding contralateral scalp needle served as the ground for LFP.

### Electrode localization

The ECoG electrode location was determined using intraoperative CT registered to the preoperative MRI with cortical surface extracted in the FreeSurfer or FastSurfer software v7 and visualized in Slicer 5.2.2. ECoG contacts over the precentral gyrus representing the primary motor cortex (M1) were selected for further analysis. On average, eight (range 1–11) ECoG bipolar contact pairs were used per patient (Table 1). For localization of the DBS lead, we used the CranialSuite software 6.3.1 (Neurotargeting) in the patient-specific (native) space, utilizing mid-commissural coordinates.

### Signal processing

#### ECoG (cEP) analysis

Data analysis was performed using custom scripts in MATLAB vR2021b (MathWorks). Raw ECoG potentials were re-referenced in a bipolar montage using adjacent contacts, aligned by stimulus start times and averaged to generate cEP (∼120 pulses for each DBS setting). The averaged signal was baseline shifted to 0 using 1 ms of data before stimulation pulse, and a smoothing function (5-point window moving average) was additionally applied (Fig. 1D). The cEPs were classified based on the temporal order of appearance and according to the peak latencies with respect to stimulation onset as previously defined^17^: EP0 (<2 ms), EP1 (2–3.5 ms), EP2 (3.5–7 ms) and EP3 (7–10 ms). The EP amplitude was defined as the voltage difference between EP peak and its preceding trough or baseline. The presence or absence of peaks and their amplitudes depended on stimulation settings while peak latencies were relatively consistent especially within the same patient.^17^

Given the large number of stimulation settings and recording channels, we developed a novel algorithm for semi-automated detection of cEP peak latencies and amplitudes. The algorithm first defined a template signal for each channel by averaging the normalized responses evoked by the highest stimulation current. Then, using the prominence and width concepts in the *findpeak* function in MATLAB, the baseline, peaks and troughs of the template were determined in a recursive and supervised manner. The algorithm then searched for the baseline and the extremum points of the evoked potential within the width of the template. All algorithm selections were visually confirmed. In cases where baseline could not be clearly identified due to the large stimulation artefact, EP1 was not defined (6% of the recordings).

The accuracy of the algorithm was confirmed by comparing with previously reported manually extracted EP1 amplitudes.^36^ The average correlation coefficient between the algorithm and manual assessment was 0.92 ± 0.04, with 5.1 ± 2.0% of algorithm detections requiring user revision. Given the large amount of data requiring visual inspection even with the semi-automated approach, we identified one best representative channel to report for EP2 and EP3 analysis (a channel with minimal noise and the largest cEP amplitudes for both anodic and cathodic stimulation, with anodic response prioritized if necessary). The EP0 was extracted manually since automated detection could not be implemented given its variable proximity to the stimulation artefact (one best channel was selected for this analysis).

#### EMG (mEP) analysis

Similar to the cEP analysis, EMG responses were aligned by stimulus start times and averaged to generate mEP for each recorded muscle in response to each stimulation setting (Fig. 1E). The presence of mEP was determined by visual inspection as a clear deflection from the baseline at expected latency (∼10 ms for facial muscles and 15–30 ms for arm muscles), and peaks and troughs were manually annotated. The mEP amplitude was defined as the largest peak-to-trough distance (mEP could be multiphasic with multiple peaks and troughs). For each patient, the best muscle was defined as the one with the most mEP responses. In most patients, this also corresponded to the muscle with the largest mEP amplitudes, and for all but three patients, it was also the channel that correlated best with EP0.

#### DLEP analysis

The LFP signals were recorded in a monopolar montage from all non-stimulating contacts on the DBS electrode. As with other EP types, stimulation times were aligned and LFP signal averaged. Given the close proximity of recording contacts to the stimulating contact, a large stimulation artefact was present. To remove the stimulation artefact and extract DLEP signal from all recording contacts, we developed a novel processing pipeline, based on fitting the stimulation artefact with a family of damped sinusoids and a post-smoothing step with a Savitzky–Golay filter. With this approach, the stimulation artefact was modelled as the compounded step response (the stimulation waveform) of the transfer function of a second-order system^37^ (Fig. 1E). The DLEP amplitude was defined as the root mean square of the artefact-free signal in the 4–20 ms post-stimulation recording window. For each stimulation setting, we calculated the average DLEP amplitude from all contacts/segments immediately adjacent (dorsal and ventral) to the stimulating contact. DLEP latency was defined as the latency of the first peak after applying the filtering.

### Pathway activation analyses

#### Recruitment curves

The recruitment curve graphically represents the relationship between the stimulation current intensity and the corresponding neuronal response (EP amplitude) while holding all other stimulation parameters constant (stimulus polarity, active contact and pulse width). The recruitment curves were constructed for each EP type, both for individual patients and on a group level. To allow comparison of EP amplitudes on a group level, EP amplitudes were normalized from 0 to 1 (the largest amplitude during 60 µs pulse width stimulation was defined as 1 since 60 µs settings were used in all patients). For mEP, the normalized values for each muscle were also averaged to obtain one mEP amplitude for each stimulation setting. For group curves, we used only stimulation settings with 60 µs pulse width (P09 excluded since 60 µs stimulation was not applied) and the bottom active contact. Due to limited experimental time, we chose to do stimulation mostly at the bottom contact since this was a ring contact (avoids having to simultaneously activate multiple segments), and it was reliable in the STN (the topmost ring contact is often dorsal to the STN). This way we could do parameter sweeps where we changed polarity, amplitude and pulse width while keeping the stimulation location constant. At least two patients had to contribute data for a specific stimulation amplitude to be included in the group analysis.

We also constructed computational modelling-based recruitment curves by plotting the percentage of activation for CSBT and HDP pathways in response to cathodic and anodic ascending currents, as reported in a previously published data set.^13^ This study used multi-compartment cable models of HDP and CSBT axons (referred to as internal capsule pathway in the original study), coupled to finite element models of the DBS electric field, to study the initiation and propagation of action potentials in these pathways. They developed a detailed representation of the human motor HDP using generative modelling methods to create axonal arbours that mimic histological reconstructions from non-human primate motor HDP reconstructions.^38^ A hundred arbourized motor HDP and CSBT fibres were converted into NEURON models^39^ with varying axon diameters. The percentage of active fibres was calculated based on action potential initiation and propagation in response to different stimulation parameters and polarities during subthalamic DBS.

#### Strength–duration curves

Given that different neuronal elements (e.g. fibres of passage and local neurons) have different strength–duration characteristics, we constructed strength–duration curves for all EP with direct neuronal origin (excluding mEP) in five patients (P11–P15). In each patient, we tested settings sweeping the pulse width from 30 to 400 µs. For each pulse width, we applied an ascending range of current amplitudes (5 on average) for both cathodic and anodic polarity. To find the activation current threshold for each pulse width, we constructed a recruitment curve for each EP measure. The stimulation current at the first inflection (knee) point of the recruitment curve was selected as the activation threshold. If the knee point was not present, we chose one EP value for both cathodic and anodic recruitment curves and defined corresponding stimulation currents as the activation current thresholds. The strength–duration curves were constructed by plotting pulse widths (duration) on the *x*-axis and activation thresholds (strength) on the *y*-axis. From each strength–duration curve, we then calculated the chronaxie metric, a time constant closely related to the time constant of the firing neuronal element.^31^ We used Weiss’s linear relationship^40,41^ between threshold charge (pulse width ∗ stimulation current) and pulse width (duration) to estimate the chronaxies. Specifically, chronaxie time was defined as the intersection with the pulse width (*x*-axis) and calculated by dividing the *y*-axis intercept value by the slope.^40,42,43^

#### Excitability metric

To compare pathway excitability during cathodic or anodic stimulation, we calculated area under the curve (AUC) of the averaged normalized group and individual patient recruitment curves. Different metrics have been used to characterize the stimulation response in prior studies including the AUC, slope of the original recruitment curve or slope of a fitted sigmoid curve.^44-47^ We chose AUC as our recruitment curve characterization metric since we did not have enough data points for each patient to reliably extract a slope or fit a sigmoid function (we could not determine if recruitment curve curves reached the saturation limit, which is necessary to calculate the slope or fit a sigmoid curve). To calculate the AUC, we used trapezoidal integration method in which the total area is first divided into smaller trapezoids between each successive data point and then summed up. For both cathodic and anodic recruitment curves, the AUC was calculated from 0 mA to the highest stimulation amplitude available for both polarities (typically 6 mA).

### Statistics

We compared EP amplitudes and latencies between paired stimulation settings (same stimulation parameters except waveform polarity). To avoid collinearity between recorded channels, we selected the best representative channel for each EP and calculated the difference between anodic and cathodic amplitudes. Then, we fit Gaussian generalized estimating equation models to the difference’s values with exchangeable correlation structure and jack-knife variance estimates.^48^ This robust approach accounts for possible correlations between observations at different settings in the same patient. Then, the intercept in these models tests the null hypothesis that there is no difference between cathodic and anodic settings. We repeated this analysis for EP latencies. We also examined EP1 in all M1 channels for each patient, in which we used Wilcoxon signed-rank tests for each patient (treating channel and setting as the unit of replication) and a Bonferroni correction to account for the number of tests in this subanalysis (i.e. number of patients). To compare the AUC of cathodic and anodic recruitment curves in individual patients, we again used non-parametric Wilcoxon signed-rank test. Similarly, we used the Wilcoxon signed-rank test to compare cathodic and anodic current threshold for HDP activation (associated with EP1). A *P*-value of <0.05 was considered statistically significant.

## Results

We recorded subdural ECoG, LFPs and EMG signals in 15 patients with Parkinson’s disease in response to low-frequency cathodic or anodic monopolar DBS in the STN (Table 1; Fig. 1). The signals were epoched and averaged to reveal evoked potentials associated with antidromic or orthodromic activation of neural pathways in the STN region. The very short-latency (<2 ms) cortical EP (EP0) and peripherally recorded mEP were a metric for activation of CSBT; short-latency (2–10 ms) cortical EP (EP1–3) for activation of HDP; and DLEP for activation of STN-GP pathway. On average, we tested 30 (range 4–62) cathodic and 20 (4–60) anodic simulation settings for each patient depending on the available intraoperative experimental time. We varied the active contact, stimulation amplitudes and stimulation pulse width, while the frequency was set to 10 Hz to allow EPs to be recorded between the pulses. We tested a total of 157 paired stimulation settings (average 10 per patient; range 4–32) meaning all stimulation parameters were the same except for the waveform polarity, allowing direct comparison of EP responses for cathodic and anodic stimulation.

### Anodic stimulation evokes less neural activity than cathodic stimulation

**Figure 2:**
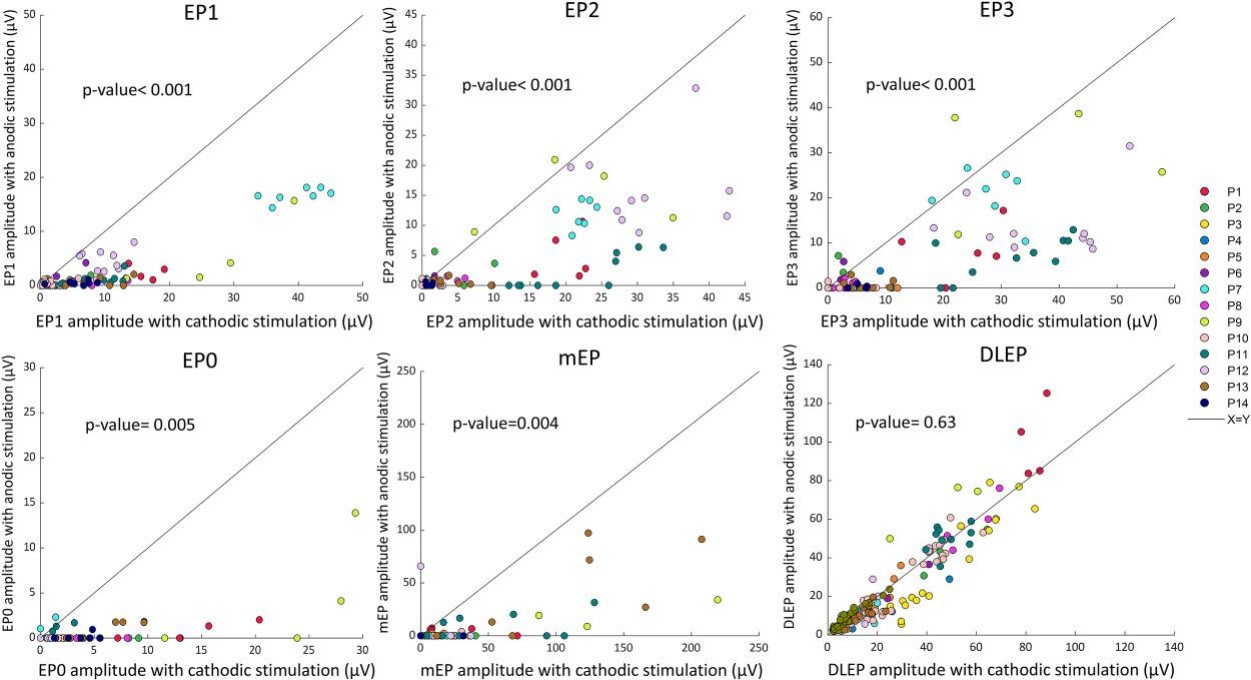
**Comparison of evoked potential amplitudes in response to cathodic and anodic stimulation (paired stimulation settings).** Each point represents EP response for one stimulation setting pair, coloured by patient. Response from one ‘best’ recording channel (largest response) is shown for clarity. The *P*-values indicate significant differences in all pairwise comparisons with most responses lying below the unity line indicating larger EP amplitudes with cathodic stimulation, except for DLEP. *P*-values are from Gaussian generalized estimating equation method. The Wald statistic value for EP0–3, mEP and DLEP was 8.08, 15.88, 12.2, 12.9, 8.24 and 0.23, respectively. *N* = 14 for EP0–3; *N* = 13 for mEP; *N* = 14 for DLEP. DLEP, DBS local evoked potential; mEP, motor evoked potential.

For EP1, EP2, EP3, EP0 and mEP, the response amplitude evoked by cathodic stimulation was significantly larger compared with the amplitude evoked by its paired anodic stimulation (Fig. 2; Supplementary Table 1). The DLEP amplitude did not significantly differ between cathodic and anodic stimulation (*P* = 0.63). The same pattern of higher amplitudes with cathodic compared with anodic stimulation held true when we inspected EP1 responses in all M1 channels (Supplementary Fig. 2) and analysed M1 channels in patients individually (Supplementary Table 2). The normalized mEP over all channels (and not only the best channel) was also consistently larger for cathodic stimulation (Supplementary Fig. 2). EP0 and mEP, both used as measures for CSBT activation, were also shown to be associated across setting within each patient with average correlation coefficient and accuracy of 65.5% ± 27.6% and 79.8% ± 16.3%, respectively (Supplementary Table 3).

To investigate whether both anodic and cathodic EP responses had the same morphology, we additionally compared the latencies of cathodic EP peaks with their paired anodic latencies (Supplementary Fig. 1; Supplementary Table 1). The latencies of cathodic and anodic peaks followed the same predefined millisecond ranges (Fig. 1). However, on average, we observed a small delay (100–500 µs) for anodic stimulation in EP2, EP3 and EP0 (*P* < 0.01), while delays for EP1, mEP and DLEP were not significant.

### Relative excitability comparing cathodic and anodic stimulation is pathway specific

**Figure 3:**
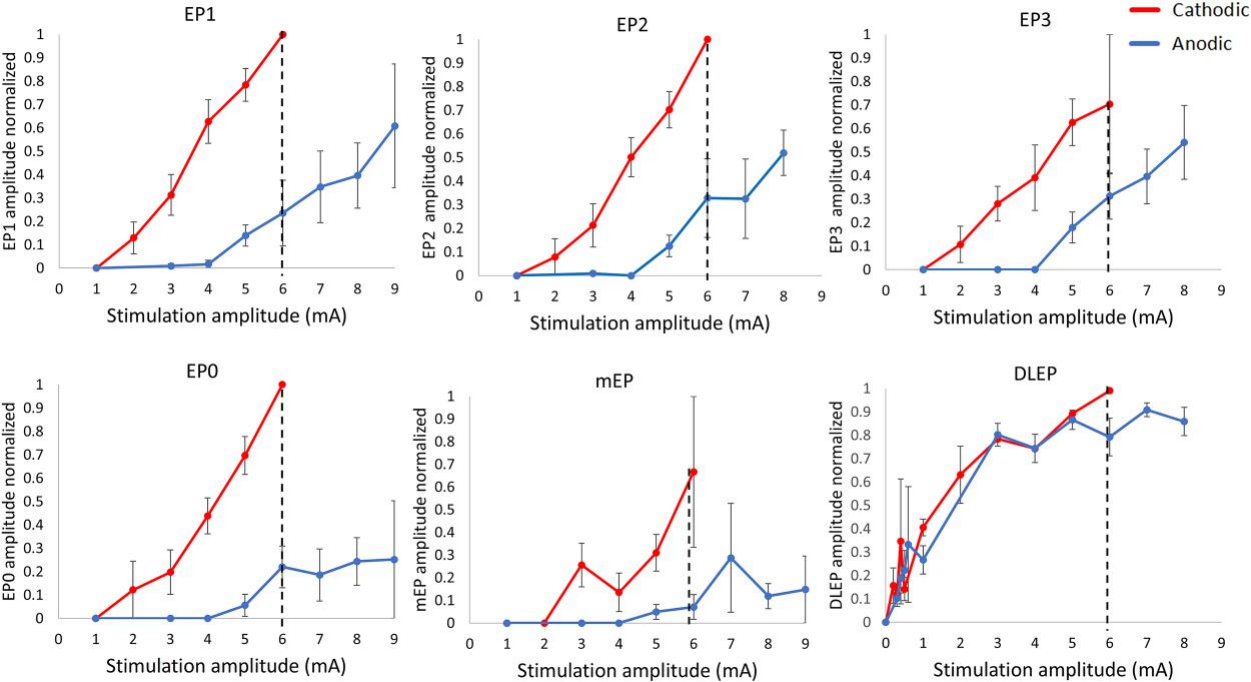
**Group recruitment curves for all EP during cathodic or anodic stimulation.** The recruitment curves were generated by averaging normalized EP amplitudes during stimulation at the ventral-most active contact and 60 µs pulse width. Error bars indicate standard error. The dotted line at 6 mA indicates the highest common stimulation amplitude up to which the AUC was calculated. Error bars indicate ± 1 SE. *N* = 13 for EP1; *N* = 12 for EP2 and 3; *N* = 13 for EP0; *N* = 12 for mEP; *N* = 13 for DLEP. DLEP, DBS local evoked potential; mEP, motor evoked potential.

To compare relative excitability of different pathways during cathodic or anodic DBS, we generated group recruitment curves using normalized EP amplitudes (Fig. 3). The AUC quantifies the degree of activation while the ratio between cathodic and anodic AUC provides a measure of relative excitability (higher ratio indicates that cathodic stimulation activates a pathway more than anodic). The highest AUC ratio was observed for EP0 and mEP at 11.8 and 12.2, respectively. Mid-range AUC ratios were measured for EP1–3 at 8.2, 6.6 and 5.3, respectively, while DLEP AUC ratio was the lowest at 1.1.

We conducted a similar analysis between AUC of cathodic and anodic recruitment curves for individual patients (Supplementary Fig. 3). The AUC values were significantly higher during cathodic compared with anodic stimulation for all EPs except DLEP (Supplementary Table 4). The ratios of average cathodic-to-anodic AUC followed the same trend as for the group recruitment curves with EP0 and mEP the highest (14.8 and 14.6), followed by EP1–3 (5.5, 4.6 and 3.9) and then DLEP (1.1). This is consistent with the group AUC analysis, and the difference between EP0 and EP1 ratios is even more pronounced.

### Experimental excitability metrics are consistent with computational modelling predictions

**Figure 4:**
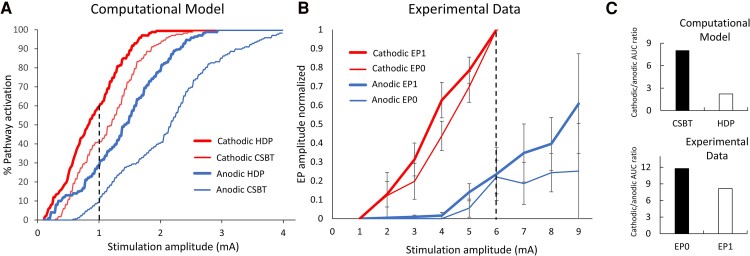
**Comparison between computational modelling and experimental recruitment curves for cathodic and anodic DBS. (A)** Recruitment curves for per cent activation of HDP (thick lines) and CSBT (thin lines) pathways from a computational modelling study (adapted from Bingham *et* al.^13^). **(B)** Recruitment curves for experimental EP1 (thick lines) and EP0 (thin lines) amplitudes, as measures for HDP and CSBT activations, respectively. The dashed line illustrates maximum current for AUC calculation. **(C)** Comparison between cathodic/anodic AUC ratio for modelling (top) and experimental data (bottom). Error bars indicate ± 1 SE. CSBT, corticospinal/bulbar tract; HDP, hyperdirect pathway.

We compared recruitment curves based on a recent STN DBS computational modelling study with the corresponding curves from our experimental data (Fig. 4). For the computational modelling curves, we reformatted data reported in Bingham *et al*.^13^ and plotted the percentage of activation for CSBT and HDP pathways in response to cathodic and anodic ascending currents (Fig. 4A). For comparison, we plotted on the same graph the recruitment curves reported in the previous section for EP0 and EP1, as respective evoked potentials associated with CSBT and HDP activation (Fig. 4B). In general, computational predictions reached full (100%) pathway activation at lower current amplitudes compared with our EP measures, which did not appear to reach saturation. To allow a comparison between modelling and experimental results, we chose a 1 mA cut-off to calculate AUC for the model since this corresponds to an ∼50% activation point. The cathodic-to-anodic AUC ratios for the CSBT and HDP were 8.05 and 2.22, respectively, for the model compared with 11.8 and 8.2 for experimental EP0 and EP1 (Fig. 4C). While the ratio values are not the same, the consistent finding between model predictions and experimental results is that the CSBT excitability metric is higher compared with the HDP excitability metric.

### Anodic stimulation may activate the same neuronal elements as cathodic stimulation

**Figure 5:**
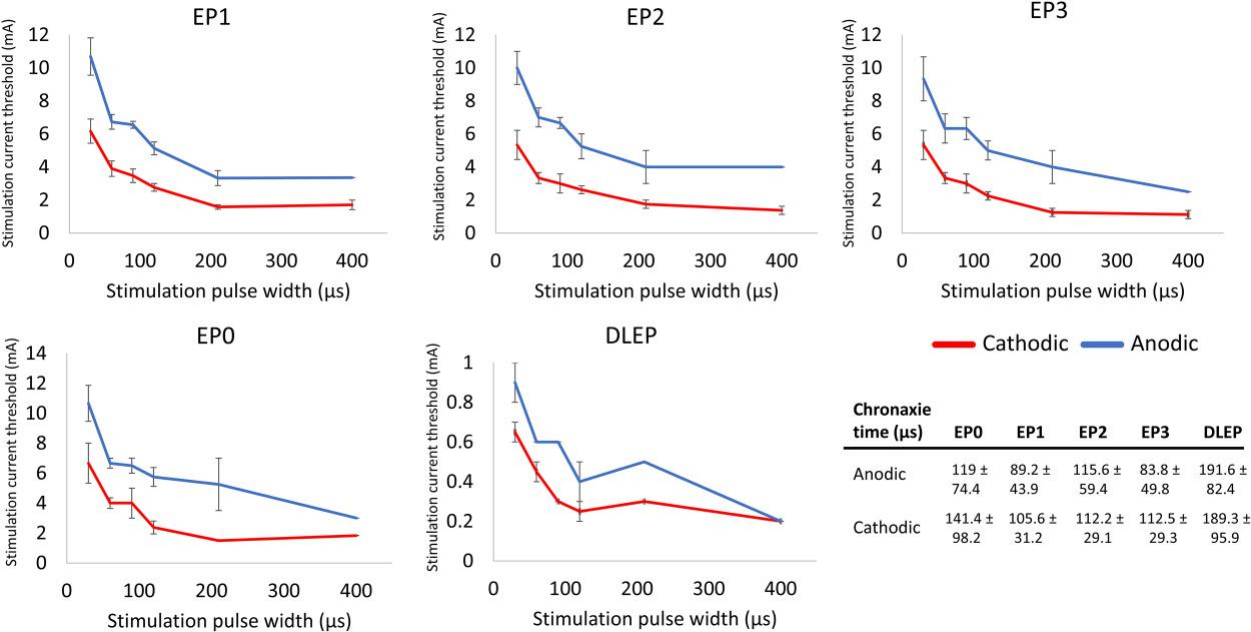
**Strength–duration curves for evoked potentials with neuronal origin.** The average chronaxie times extracted from individual patient’s strength–duration curves are reported in the table. *N* = 4 for cEP; *N* = 2 for DLEP (DLEP 400 µs point is based on 1 patient only). Error bars indicate ± 1 SE. *N* = 4 for EP0–3; *N* = 2 for DLEP (DLEP 400 µs point is based on one patient only). DLEP, DBS local evoked potential.

In order to investigate which neuronal elements were first activated during stimulation, for a subset of patients (*N* = 4 for cEP; *N* = 2 for DLEP), we constructed strength–duration curves for all evoked potentials with direct neuronal origin (all except mEP) and calculated corresponding chronaxie times (Fig. 5). This required varying pulse width from 30 to 400 µs and testing on average 5 stimulation amplitudes to find an approximate EP activation threshold for each pulse width and for each polarity. For all evoked potentials, the calculated chronaxie values for both cathodic and anodic stimulations were comparable and within the same range of 80–140 µs for cEP and ∼190 µs for DLEP (Fig. 5). Formal statistical testing was not feasible due to the low number of data points, but nonetheless chronaxie values in this range are consistent with activation of myelinated fibres.^4,49^ These chronaxie values are also consistent with biophysical models of STN neuron activation with DBS.^50^

### Anodic stimulation requires higher current amplitude for similar degree of activation as cathodic stimulation

Given that anodic stimulation was consistently less efficient in activating HDP and CSBT pathways, we asked what current amplitude is needed for anodic stimulation to achieve the same activation as cathodic stimulation. Using individual patient recruitment curves (Supplementary Fig. 3), we extracted the current stimulation amplitude needed to evoke EP1 of 1 µV as a threshold for HDP activation. We used data from seven patients who had sufficiently detailed recruitment curves (3 or more data points) for both polarities and current thresholds were averaged across all M1 channels. On average, the cathodic current threshold for HDP activation was 2.46 ± 0.8 mA compared with 5.26 ± 1.4 mA for anodic stimulation (*P* = 0.031). The average anodic/cathodic current ratio was 2.2 ± 0.44, indicating that more than twice the anodic current was needed to activate the HDP (Supplementary Table 5).

## Discussion

We compared evoked potentials attributed to activation of clinically relevant pathways in the subthalamic region in response to anodic and cathodic DBS. We studied intracranial and peripheral (muscle) responses in 15 patients with Parkinson’s disease. By focusing on stimulus polarity, our goal was to elucidate biophysical mechanisms that underlie electrical stimulation in the human brain. Our key observations are (i) subcortical cathodic and anodic stimulations evoke similar responses in the cortex, subcortically and peripherally, but anodic responses are generally smaller for the same current amplitude; (ii) relative efficiency of anodic stimulation compared with cathodic varies by EP measure meaning that anodic stimulation is the least efficient in activating CSBT (EP0/mEP), moderately efficient in activating the HDP (EP1/EP2/EP3), and as efficient as cathodic in activating STN-GP pathway (DLEP); (iii) cathodic and anodic stimulations have comparable chronaxie times for all EP measures; (iv) relative excitation efficiency for experimental EP0 and EP1 markers is consistent with computational studies modelling activation of CSBT and HDP pathways, respectively; (v) anodic stimulation requires approximately twice as much current as cathodic to achieve the same degree of EP1 (HDP) activation.

### Cathodic and anodic stimulations activate the same neural pathways

EP measures that we utilized in this study have previously been attributed to activation of specific pathways in the subthalamic region.^16-26,28,29,51-53^ In this study, we demonstrate that the morphology of evoked responses is similar during cathodic and anodic stimulation suggesting that the same pathways are activated regardless of stimulus polarity. Specifically, we observed the same number, shape and general timing of peaks in the cortex, subcortically and peripherally; however, with anodic stimulation, the peaks were smaller and, in the cortex, sometimes delayed by up to 0.5 ms.

**Figure 6:**
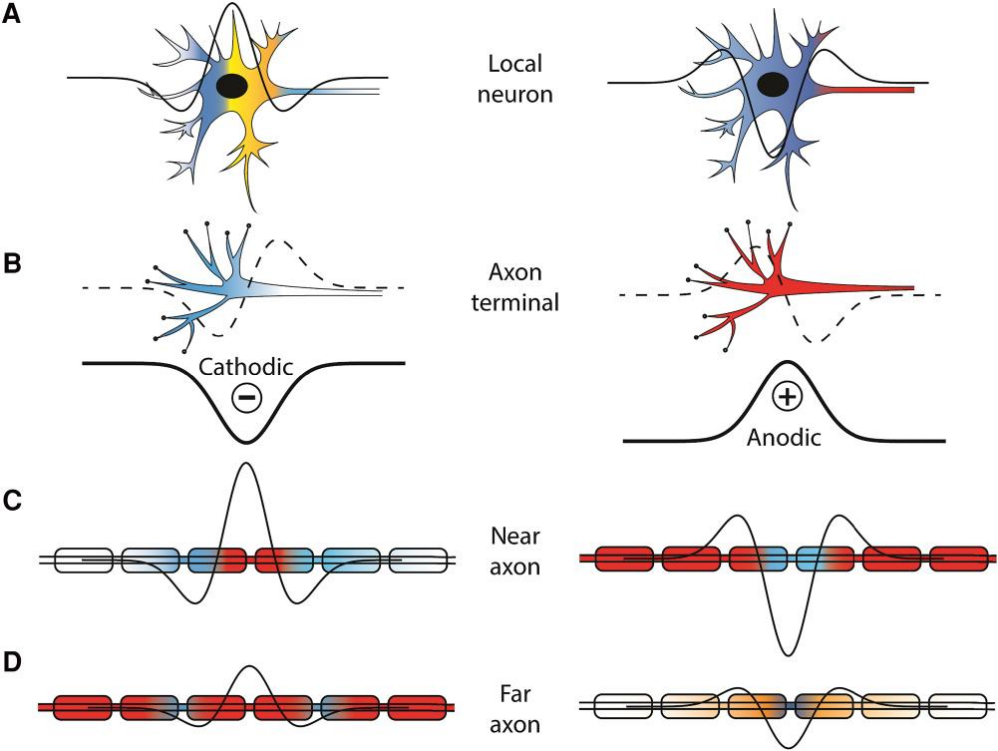
**Comparison between activation of different neuronal compartments in response to cathodic (left) and anodic (right) stimulation.** Depolarization is depicted in warm colours while hyperpolarization is in green. Thick line shows extracellular potential around monopolar cathode (left) or anode (right) decaying with distance from the electrode. Action potential initiates in the region with over-the-threshold depolarization. When cell body of a local neuron is the closest compartment to the electrode **(A)**, activation occurs at the axon hillock as sufficient direct depolarization of cell body is difficult to achieve with extracellular stimulation.^4,58^ Activation of axon terminals **(B)** is regulated by the first spatial derivative of the electric potential, which is symmetric for cathodic and anodic stimuli (dashed line). Activation of passing axons (including the axon hillock) is driven by the second spatial derivative of the electric potential (thin line; a.k.a. the activating function). Axons closest to the electrode **(C)** may not be activated by cathodic stimulation due to strong hyperpolarization blocking the propagation of action potential (known as the ‘anodal surround’^4^), while those farther away **(D)** are most excitable by cathodic stimulus. For anodic stimulation, the electric potential along a passing axon is reversed compared with cathodic so that there is a large hyperpolarization near the electrode and small depolarizations at the flanking regions. As a result, a larger anodic stimulus is needed to achieve suprathreshold activation at the depolarized flanking regions. Note that activation of neural elements by extracellular electric field does not depend solely on the value of its first or second spatial derivative but rather it is driven by the precise timing and spatial distribution of induced currents exiting and entering neural compartments and leading to their depolarization or hyperpolarization. This is a dynamic process that cannot be easily captured in a schematic, but the purpose here is to provide a visual guide highlighting relevant biophysical concepts.

From physiological studies in the peripheral and central nervous system, it is known that stimulating an axon fibre passing by a monopolar electrode requires less cathodic current than anodic.^4,49,54^ This is because the electric field for cathodic stimulation generates a large depolarization near the electrode with small hyperpolarization flanking it. In cable theory, this is expressed mathematically as the ‘activating function’ stating that membrane polarization and activation of a straight axon (a cable) depends on the second-order spatial derivative of the extracellular electric potential (Fig. 6).^55-57^

### Cathodic and anodic stimulation may activate the same neural elements

To identify the primary neural elements related to activation of different pathways, we constructed strength–duration curves for all EP measures and extracted chronaxie time constants. Smaller chronaxie values indicate more excitable neural elements.^59^ Previous studies have estimated the chronaxie values for large, myelinated axons to be ∼30–200 ms, for small axons ∼200–700 ms and for cell bodies and dendrites ∼1–10 ms.^4,40,43^ In our study, the chronaxies for all EPs were comparable for cathodic and anodic stimulation and fell within the range associated with axonal activation. We found that the average chronaxie values for DLEP (∼200 µs) were longer, which might be an indicator of a mixture of other neuronal elements being activated for that pathway, such as terminal synapses or axon hillock.^4^

### Efficiency of cathodic compared with anodic stimulation is pathway specific

One key finding of this study is that the relative activation efficiency (amount of activation per unit of current) of cathodic compared with anodic stimulation varies by the pathway. This has important implications regarding how precisely pathway activation initiates when extracellular stimulation is applied subcortically. Similar chronaxies suggest that activation occurs in the same axonal element regardless of polarity, but differences in relative efficiencies suggest that different axonal elements may be involved for different pathways. We propose that differences in relative activation efficiency arise from different pathway distances from the stimulating electrode and variations in which neural elements are exposed to the electric field given the specific local anatomy of this brain region.

The CSBT is composed of large, myelinated fibres that pass parallel to the stimulating electrode usually at some distance from the electrode, and the goal of clinical STN DBS is to avoid CSBT activation and associated side effects. Since the axons are passing by the electrode, the activation of CSBT is driven by the second spatial derivative of the extracellular potential in which case anodic stimulation is much less efficient.^4^ This was demonstrated in our study for EP0 and mEP, which both measure CSBT activation (antidromically for EP0 and orthodromically for mEP). Similar observations were made recently by Campbell *et al*.^25^ where anodic monopolar STN DBS resulted in much smaller mEP compared with cathodic.

The HDP is a direct input from the cortex to the STN formed primarily by thin collaterals from corticofugal axons passing through the internal capsule and continuing towards the brainstem, although some may be direct connections.^38,60,61^ In our study, anodic stimulation was relatively more efficient at activating the HDP compared with CSBT (lower cathodic/anodic activation ratio) (Figs 3 and 4). This suggests that HDP activation involves activation of axonal elements that differ from the distant, large, myelinated passing fibres. Given the anatomical location of the stimulating electrode in the STN, the HDP axons are at varying distances from the electrode as they enter the same target area. Paradoxically, the axons that pass the closest to the electrode are not activated by suprathreshold cathodic stimulation due to anodal surround phenomenon.^4^ That means that anodic stimulation would gain some advantage in this situation over cathodic (Fig. 6C and D). Furthermore, the activation of axon terminals is predicted to be driven by the first spatial derivative of the extracellular voltage because of the biophysical properties of the final segment of the multi-compartment cable model.^15,57,62,63^ Consequently, axon terminals are equally likely to be activated by anodic or cathodic stimulation given the symmetric spatial distribution of the electric field (Fig. 6B). As a result, we propose that HDP activation is likely achieved by exciting passing axons at varying distances from the electrode as well as by activating terminal axons synapsing in the target region.^64^

The DLEP is thought to result from activation of STN-globus pallidus externa (GPe) projections inducing resonant activity between these two nuclei that are reciprocally connected with excitatory (STN-to-GPe) and inhibitory (GPe-to-STN) projections.^26,65^ In contrast to all the other EPs that we studied, the DLEP anodic excitability was very similar to DLEP cathodic. There are two neural elements where anodic activation has an advantage over cathodic: terminal synapses and cell bodies (Fig. 6A and B). The terminal axons present in the STN consist of excitatory cortical projections (HDP) and inhibitory afferents from the GPe, which together can generate DLEP activity.^26^ The STN local neurons, which project to the pallidum, are also directly activated by the stimulating electrode.^8^ Neurons with cell bodies in proximity to the active electrode can be activated with lower thresholds using anodic stimulation due to the depolarization of the axon.^4,57,58,66,67^ As a result, the activation of STN-GPe pathway and DLEP generation is likely due to combined activation of terminal synapses converging in the STN and activation of local STN projection neurons.

### Experimental findings confirm some biophysical DBS model predictions

For several decades, computational studies utilizing multi-compartment neuron models have predicted that neural activation thresholds depend on the spatial position and orientation of the neuron relative to the electrode, in addition to the electrical properties such as type and distribution of ion channels, as well as capacitance and conductance of neural compartments.^55,57,66^ These models have been instrumental in helping explain how extrinsic stimulation, such as DBS, engages with the nervous system.^26,34,68-71^

Today, the most advanced computational DBS models utilize representations of individual cortical projection neurons with complex axonal arbours that mimic histological reconstructions to predict activations of HDP and CSBT pathways.^13^ This level of detail is important because activation thresholds depend on neuronal morphology.^15^ Compared with cathodic monopolar stimulation, detailed models predict higher activation thresholds with anodic for both pathways, as well as the longer conduction latencies to the cortex and within the STN.^13^ Furthermore, the model predicts that relative efficiency of anodic stimulation is pathway specific (anodic is relatively efficient at activating HDP but less efficient for CSBT), which is consistent with our experimental data. This phenomenon can be attributed to the greater distance of the CSBT from the stimulating electrode compared with the fibres in the HDP and likely involvement of terminal axons in HDP activation.^64^

A recent modelling study by Anderson *et al*.^14^ has suggested that anodic stimulation should be more efficient than cathodic when activating neurons orthogonal to the electrode (leaving or approaching the electrode so that terminal synapse or cell body is closest to the electrode) compared with passing axons. The authors have predicted that because HDP approaches the DBS electrode orthogonally, the HDP activation will be more efficient during anodic compared with cathodic stimulation. Our experimental data do not support more efficient activation of the HDP, but rather, the STN-GP pathway (DLEP marker) was similarly excitable by anodic and cathodic stimulation. So, while the modelling principles are valid, we suggest that they apply to a different pathway.

### Differences in pathway excitability shed light on clinical DBS mechanisms

**Figure 7:**
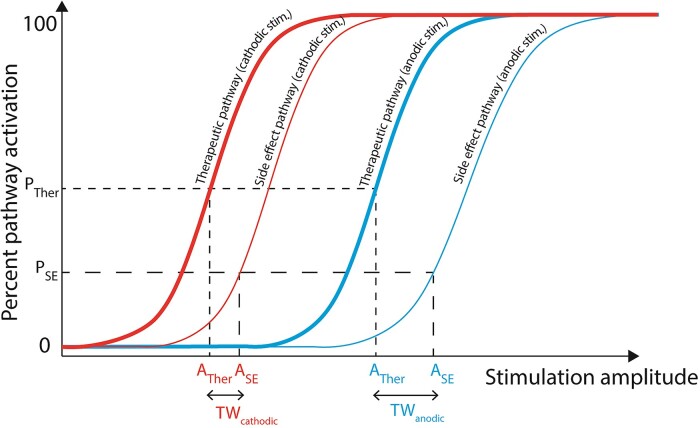
**A hypothesized relationship between recruitment curves and clinical thresholds for activation of therapeutic (thick line) and side effect (thin line) pathways in response to cathodic and anodic stimulation.** A, amplitude; P, per cent; SE, side effect; Ther, therapeutic; TW, therapeutic window.

There are many pathways coursing through the STN region, and it is still unclear which pathways are responsible for therapeutic benefit in STN DBS.^72^ Specifically, there has been an ongoing debate regarding the relative contributions of the HDP and subthalamo-pallidal pathway (STN projections to globus pallidus interna and GPe are part of the indirect pathway).^73^ It is widely accepted that CSBT is the pathway causing motor side effects.^11,24^ During clinical DBS programming, two thresholds are typically determined by gradually increasing stimulation amplitude. The therapeutic threshold represents stimulation amplitude at which therapeutic benefit emerges (e.g. tremor reduction) while side effect threshold is the stimulation amplitude where adverse effects are observed (e.g. muscle contractions). The therapeutic window is the difference between the therapeutic and side effect thresholds (Fig. 7). Chronic stimulation is typically applied at electrode contact with the largest therapeutic window so it is desirable to devise stimulation strategies that can widen the therapeutic window.

Two recent clinical studies have established that monopolar anodic DBS can achieve similar (or even greater) clinical benefit as cathodic, but it requires higher current amplitudes.^6,7^ Side effects were also induced at higher anodic thresholds, but overall, the therapeutic window was wider for anodic stimulation. In our experiments, activation of HDP required more anodic current while activation of STN-GP did not, suggesting that HDP may be the pathway responsible for therapeutic benefit observed in clinical studies. If the STN-GP pathway was the main driver of clinical benefit, then we would expect therapeutic thresholds to be similar for anodic and cathodic DBS. CSBT activation required more anodic current in clinical studies, which is consistent with our experimental findings.

Our experiments were not designed to assess therapeutic benefit, but we can compare pathway activation thresholds in our study to threshold amplitudes for therapeutic efficacy in prior studies. We determined that the average cathodic threshold to activate the HDP was 2.46 ± 0.8 mA compared with 5.26 ± 1.4 mA for anodic, which is similar to therapeutic benefit amplitudes reported in clinical studies. In Kirsch *et al*.,^6^ therapeutic benefit thresholds were 1.99 ± 1.37 mA for cathodic and 3.36 ± 1.58 mA for anodic, while Soh *et al.*^7^ reported 3.8 ± 1.6 and 4.9 ± 2.1 mA, respectively. In our experiments, it took 2.2 times more anodic current than cathodic to achieve the same HDP activation (anodic to cathodic threshold ratio), while in clinical studies, the ratio to achieve therapeutic benefit was 1.3–1.7. Overall, our findings suggest that activation of the HDP may be involved in therapeutic effects of STN DBS, but contribution of other pathways in the target region cannot be ruled out.

### Limitations

Our study has several limitations: (i) the number of stimulation settings examined varied between patients and the recruitment and strength–duration curves had coarse resolution. This was due to variations in the available experimental time in the operating room. Nonetheless, patients showed consistent responses, which allowed us to combine results for group analysis; (ii) stimulation waveforms had a low-amplitude second recharge phase of opposite polarity as required for safe delivery of electrical stimulation in human subjects.^74^ It is possible that the recharge phase contributed to neural activation although we were careful to exclude stimulation settings where the recharge phase amplitude would be above the activation threshold, and in several patients, we utilized an analogue stimulation protocol to prolong the second phase for strength–duration curve calculations; (iii) DLEP thresholds required for strength–duration curve calculations were difficult to determine (thresholds were consistently below 1 mA and recruitment curves noisy in some patients) resulting in fewer data points; (iv) we do not know if Parkinson’s disease alters pathway excitability and whether our findings are generalizable to healthy state or other conditions where STN DBS is used clinically; (v) change in EP amplitudes over time is a potential concern, but our focus was on the acute response and the same stimulation duration was used for both stimulation polarities. A prior study measuring antidromic cortical activation in non-human primates with STN DBS showed that neural responses were stable at 50 s for low (15 Hz) frequency stimulation^75^; (vi) our experiments used individual pulses delivered at low frequency (10 Hz), while clinical DBS employs high frequency (∼130 Hz). While a previous study has shown that EP1 amplitude does not depend on cathodic stimulation frequency,^17^ it remains to be tested whether pathway-specific relative efficiency holds for high frequencies and prolonged stimulation durations. A future post-operative clinical evaluation coupled with delivering stimulation settings of different polarity could enhance clinical relevance of our findings; and (vii) differences in evoked potential amplitudes between the patients (Supplementary Fig. 3) are likely due to variations in DBS lead and ECoG strip locations with respect to local anatomy. Nonetheless, normalization allowed us to perform group analyses. A complementary spatial analysis in the future, linking the locations of lead and ECoG contacts and estimating pathway activation, could help explain individual patient differences.

## Conclusions

We provide experimental evidence that relative efficiency of cathodic and anodic stimulation varies for different pathways so that anodic stimulation was the least efficient in activating CSBT, moderately less efficient in activating the HDP, and as efficient as cathodic in activating STN-GP pathway. Additionally, we demonstrated that cathodic and anodic stimulations activate the same subcortical pathways in the STN region. Our experiments confirm biophysical model predictions regarding neural activations in the central nervous system and provide evidence that stimulus polarity has differential effects on passing axons, terminal synapses and local neurons. Comparison of experimental results with clinical DBS studies provides further evidence that the HDP may be involved in the therapeutic mechanisms of DBS.

## Supplementary Material

fcaf006_Supplementary_Data

## Data Availability

The data that support the findings of this study are available from the corresponding author, upon reasonable request.
