# Supplementary material for: Neural pathway activation in the subthalamic region depends on stimulation polarity
Source: Brain Commun. 2025 Jan 21;7(1):fcaf006. doi: 10.1093/braincomms/fcaf006 (PMC11839843; doi:10.1093/braincomms/fcaf006)
Supplement: fcaf006_Supplementary_Data [file fcaf006_supplementary_data.docx]

# Supplementary Materials

## Stimulation waveform

The most common stimulation pulse width was 60 us so the second phase was typically 8 times longer with 1/8 of the amplitude of the first phase to maintain charge balance between phases. Because of the 500 us NeuroOmega limit for each phase duration, stimulation settings with pulse widths longer than 60 us had their second phase duration limited to 480 us and second phase amplitude adjusted to maintain charge balance. Consequently, for some stimulation settings, the amplitude of the second phase could be large and might contribute to neural activation. We, therefore, excluded from analysis any stimulation settings where second phase amplitude was over the EP activation threshold (400 us anodic settings for P11, P12, and P13 and 400 us cathodic settings for P13). Because long pulse width settings were necessary to construct the strength duration curves described below, in two patients (P14, P15) we utilized the analog stimulation feature of NeuroOmega where a waveform of arbitrary shape (including phase duration) can be defined. For these stimulation settings, the duration of the second phase was variable depending on the first phase pulse width, and up to 10ms.


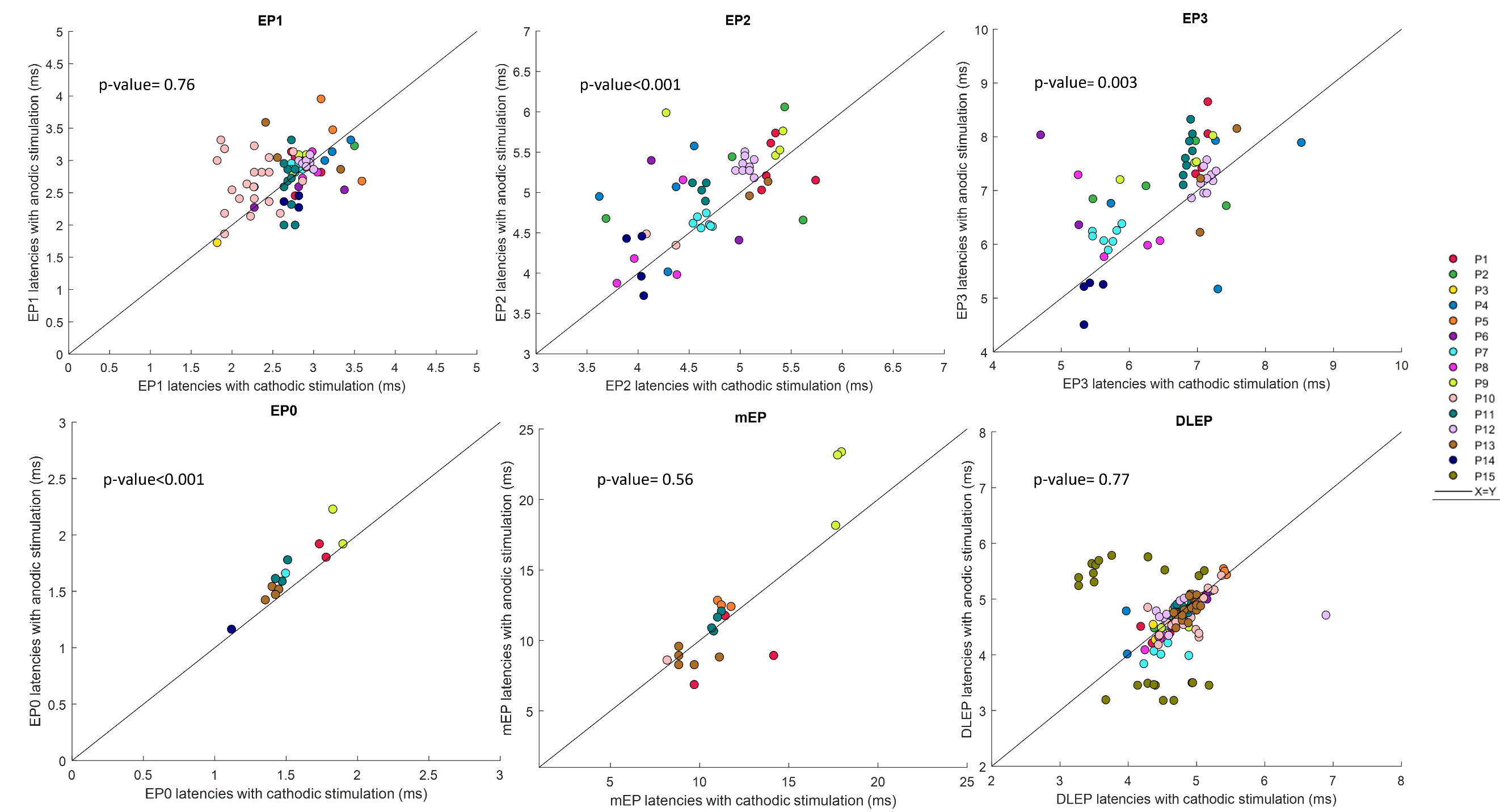


**Supplementary Figure 1. Comparison of evoked potential peak latencies in response to cathodic and anodic stimulation (paired stimulation settings).** Each point represents EP response for one stimulation setting pair, colored by patient. Response from one ‘best’ recording channel (largest response) is shown for clarity. The p-values indicates significant differences in EP0, EP2 and EP3 and DLEP pairwise comparisons with most responses lying over the unity (x=y) line indicating delayed EP peaks with anodic stimulation. N=14 for EP0-3; N=13 for mEP; N=14 for DLEP. P-values are from Gaussian generalized estimating equation method.

**Supplementary Table 1. Comparison of average evoked potential amplitudes and latencies in response to cathodic and anodic paired stimulation** (all stimulation parameters were the same except stimulus polarity). P-values are from Gaussian generalized estimating equation method.

| Mean ± SD | EP1 | EP2 | EP3 | EP0 | mEP | DLEP |
| --- | --- | --- | --- | --- | --- | --- |
| Cathodic amplitude (µV) | 8.506 ± 10.716 | 8.593 ± 11.711 | 11.654 ± 15.200 | 2.800 ± 5.373 | 20.556 ± 42.004 | 27.860 ± 23.149 |
| Anodic amplitude (µV) | 2.300 ± 4.429 | 3.314 ± 6.048 | 4.783 ± 8.435 | 0.315 ± 1.412 | 5.562 +16.715 | 26.107 ± 25.764 |
| p-value | <0.001 | <0.001 | <0.001 | 0.005 | 0.004 | 0.63 |
| Cathodic latency (ms) | 2.702 ± 0.388 | 4.729 ± 0.528 | 6.537 ± 0.807 | 1.530 ± 0.220 | 11.669 ± 3.032 | 4.698 ± 0.458 |
| Anodic latency (ms) | 2.801 ± 0.385 | 4.968 ± 0.553 | 6.964 ± 0.922 | 1.665 ± 0.270 | 11.995 ± 4.694 | 4.690 ± 0.516 |
| p-value | 0.76 | <0.001 | 0.003 | <0.001 | 0.56 | 0.77 |


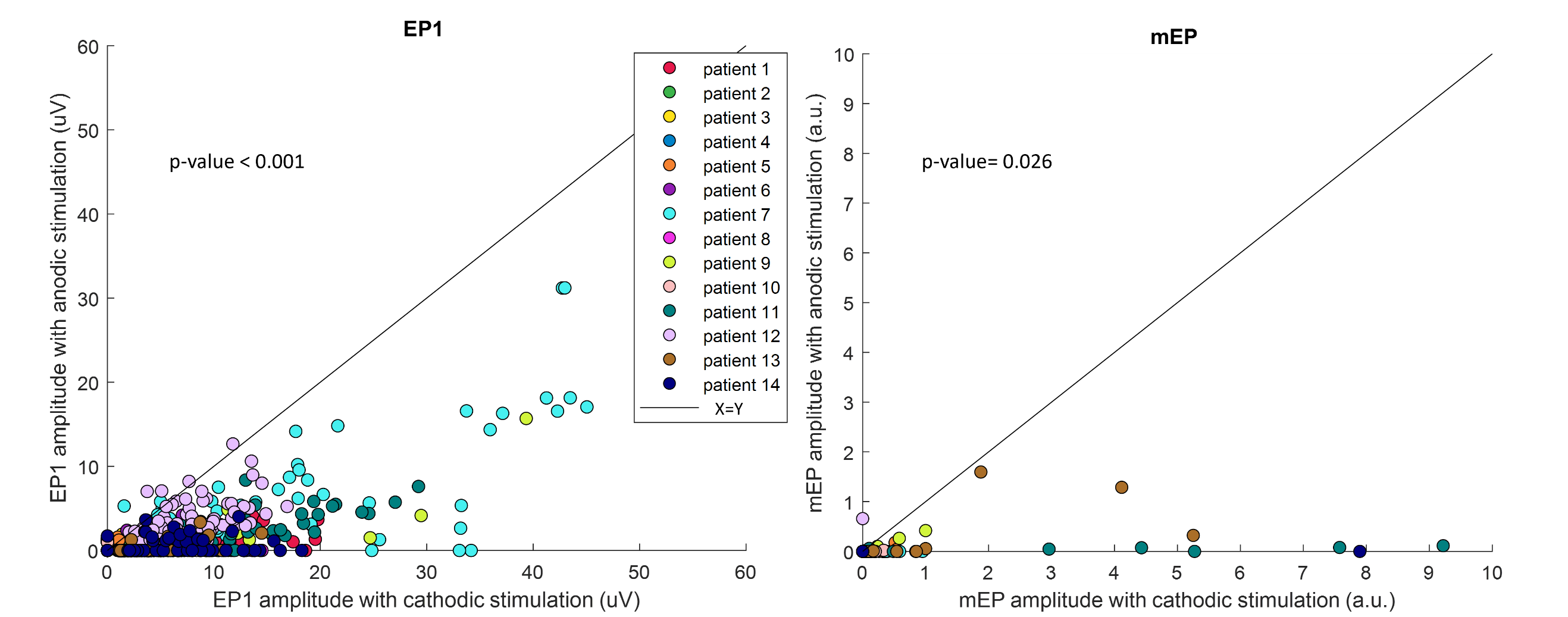


**Supplementary Figure 2. Expanded analysis of EP1 and mEP responses for paired cathodic and anodic stimulation settings.** Left: Comparison of cathodic and anodic EP1 amplitudes in all M1 channels. Each point represents EP1 amplitude in response to cathodic its matched anodic stimulation in one M1 channel. Right: Comparison of cathodic and anodic mEP amplitude with average of normalized amplitude over all EMG channels (muscles). Normalized amplitude is sometimes larger than 1, typically for settings with pulse widths longer than 60us because the highest 60 us setting was normalized to 1 for each muscle in each patient. N=14 for EP1; N=13 for mEP. P-values are from Gaussian generalized estimating equation method. The Wald statistics value for EP1 and mEP was 23.2 and 4.98, respectively.

**Supplementary Table 2. Statistical comparison of cathodic and anodic EP1 amplitudes in all M1 channels and by patient.** Adjusted p-values are from Bonferroni correction analysis.

|  |  | Number of M1/total ECoG channels | Number of paired DBS settings | Average EP1 amplitude for cathodic stimulation | Average EP1 amplitude for cathodic stimulation | Adjusted p-value |
| --- | --- | --- | --- | --- | --- | --- |
|  | P01 | 9 / 26 | 5 | 10.18 ± 4.89 | 0.80 ± 1.18 | **< 0.001** |
|  | P02 | 10 / 26 | 4 | 3.79 ± 3.33 | 0.52 ± 0.69 | **0.009** |
|  | P03 | 11 / 26 | 18 | 0.69 ± 0.95 | 0.05 ± 0.30 | **0.005** |
|  | P04 | 9 / 26 | 4 | 0.83 ± 1.14 | 0.10 ± 0.33 | **0.027** |
|  | P05 | 2 / 5 | 6 | 1.08 ± 1.30 | 0.34 ± 0.61 | 2.078 |
|  | P06 | 1 / 5 | 6 | 2.73 ± 2.82 | 1.38 ± 1.72 | 3.500 |
|  | P07 | 10 / 26 | 7 | 16.61 ± 12.96 | 6.28 ± 6.61 | **< 0.001** |
|  | P08 | 3 / 5 | 4 | 5.72 ± 4.66 | 0.08 ± 0.27 | **0.007** |
|  | P09 | 2 / 5 | 4 | 17.83 ± 12.05 | 3.69 ± 5.16 | 0.109 |
|  | P10 | 6 /26 | 24 | 0.41 ± 1.15 | 0.08 ± 0.29 | **0.003** |
|  | P11 | 7 / 26 | 10 | 10.07 ± 6.80 | 1.64 ± 2.10 | **< 0.001** |
|  | P12 | 9 / 26 | 10 | 7.72 ± 3.22 | 3.31 ± 2.34 | **< 0.001** |
|  | P13 | 8 / 26 | 8 | 4.75 ± 3.28 | 0.27 ± 0.67 | **< 0.001** |
|  | P14 | 7 / 26 | 8 | 7.08 ± 4.78 | 0.82 ± 1.11 | **< 0.001** |

P15 did not have an ECoG electrode.

**Supplementary Table 3.** T**he associations between EP0 and mEP across settings within each patient.**

|  | P01 | P02 | P03 | P04 | P05 | P06 | P07 | P08 | P09 | P10 | P11 | P12 | P13 | P14 |
| --- | --- | --- | --- | --- | --- | --- | --- | --- | --- | --- | --- | --- | --- | --- |
| Accuracy | 0.90 | 1.00 | 0.92 | 0.88 | 0.50 | 0.92 | 0.71 | NaN | 0.88 | 0.90 | 0.75 | 0.65 | 0.86 | 0.50 |
| Correlation coef. | 0.26 | 0.98 | 0.56 | NaN | 0.76 | 0.92 | 0.43 | NaN | 0.87 | 0.90 | 0.71 | 0.13 | 0.83 | 0.51 |
| p-value | 0.475 | << 0.01 | <<0.01 | NaN | 0.011 | <<0.01 | 0.121 | NaN | <0.01 | <<0.01 | <<0.01 | 0.598 | <<0.01 | 0.089 |

**Supplementary Table 4. Comparison between area under the curve (AUC) of cathodic and anodic recruitment curves for individual patients.**

|  | mEP | | EP0 | | EP1 | | EP2 | | EP3 | | DLEP | |
| --- | --- | --- | --- | --- | --- | --- | --- | --- | --- | --- | --- | --- |
|  | cathodic | anodic | cathodic | anodic | cathodic | anodic | cathodic | anodic | cathodic | anodic | cathodic | anodic |
| P01 | 2.017 | 0.036 | 1.457 | 0.213 | 1.951 | 0.271 | 1.539 | 0.212 | 1.600 | 0.162 | 2.885 | 2.969 |
| P02 | 1.204 | 0.000 | 1.317 | 0.000 | 1.080 | 0.160 | 1.606 | 0.271 | 0.910 | 0.479 | 2.957 | 2.486 |
| P03 | 0.461 | 0.000 | 0.735 | 0.000 | 0.923 | 0.000 | 0.278 | 0.000 | 0.000 | 0.000 | 2.380 | 1.340 |
| P04 | 0.000 | 0.000 | 0.882 | 0.000 | 0.246 | 0.000 | 0.000 | 0.000 | 0.514 | 0.000 | 1.827 | 1.299 |
| P05 | 2.090 | 0.322 | 0.500 | 0.000 | 0.276 | 0.020 | 1.000 | 0.000 | 1.210 | 0.000 | 2.833 | 3.006 |
| P06 | 0.342 | 0.000 | 0.433 | 0.000 | 1.236 | 0.115 | 0.598 | 0.000 | 0.438 | 0.000 | 3.685 | 3.505 |
| P07 | 0.218 | 0.000 | 2.500 | 0.000 | 2.347 | 0.919 | 1.775 | 0.812 | 1.998 | 1.452 | 2.039 | 2.500 |
| P08 | NaN | NaN | 2.500 | 0.000 | 2.500 | 0.171 | 1.988 | 0.295 | 2.227 | 0.574 | 2.331 | 2.500 |
| P09 | NaN | NaN | NaN | NaN | NaN | NaN | NaN | NaN | NaN | NaN | NaN | NaN |
| P10 | 0.412 | 0.000 | 1.000 | 0.000 | 1.518 | 0.063 | 0.132 | 0.000 | 0.504 | 0.000 | 2.923 | 2.258 |
| P11 | 0.955 | 0.124 | 1.685 | 0.529 | 1.988 | 0.281 | 2.031 | 0.000 | 2.322 | 0.244 | 3.790 | 2.857 |
| P12 | 0.791 | 0.114 | 2.230 | 0.217 | 2.744 | 1.764 | 2.335 | 1.374 | 2.298 | 0.811 | 1.965 | 2.363 |
| P13 | 0.771 | 0.038 | 1.247 | 0.126 | 1.820 | 0.105 | 1.854 | 0.309 | 0.999 | 0.150 | 4.737 | 3.896 |
| P14 | 0.000 | 0.000 | 1.044 | 0.098 | 2.594 | 0.020 | NaN | NaN | NaN | NaN | NaN | NaN |
| P15 | NaN | NaN | NaN | NaN | NaN | NaN | NaN | NaN | NaN | NaN | 3.353 | 3.233 |
|  |  |  |  |  |  |  |  |  |  |  |  |  |
| mean | 0.77 | 0.05 | 1.35 | 0.09 | 1.63 | 0.30 | 1.26 | 0.27 | 1.25 | 0.32 | 2.90 | 2.63 |
| sd | 0.70 | 0.10 | 0.70 | 0.16 | 0.84 | 0.50 | 0.82 | 0.42 | 0.82 | 0.45 | 0.83 | 0.75 |
| p-value | 0.0020 | | 0.0002 | | 0.0002 | | 0.0010 | | 0.0010 | | 0.0942 | |
| ratio | 14.612 | | 14.826 | | 5.456 | | 4.624 | | 3.880 | | 1.102 | |

**Supplementary Figure 3. Individual patient recruitment curves for all EP during cathodic (red) or anodic (blue) stimulation.** Stimulation was in the bottom (most ventral) DBS contact with 60µs pulse width (100µs for P9). The recording shown is from the ‘best’ channel. For DLEP, stimulation was in the contact that resulted in the largest DLEP response when amplitude-titration data was available for more than one contact.

**Supplementary Table 5.** **Comparison of anodic vs cathodic current thresholds for HDP activation** (EP1 amplitude > 1uV)

|  |  | | |
| --- | --- | --- | --- |
|  | cathodic (mA) | anodic (mA) | Ratio |
| P01 | 1.29 | 3.76 | 2.91 |
| P02 | 2.75 | 5.98 | 2.18 |
| P04 | 1.9 | 3 | 1.58 |
| P11 | 2.3 | 5.6 | 2.43 |
| P12 | 2.16 | 5.20 | 2.41 |
| P13 | 3.69 | 6.69 | 1.81 |
| P14 | 3.14 | 6.58 | 2.10 |
|  |  |  |  |
| mean | 2.46 | 5.26 | 2.20 |
| std | 0.80 | 1.40 | 0.44 |
